# Total Pain and Fear of Recurrence in Post-Treatment Cancer Patients: Serial Mediation of Psychological Flexibility and Mentalization and Gender Moderation

**DOI:** 10.3390/jcm13071974

**Published:** 2024-03-28

**Authors:** Dariusz Krok, Ewa Telka, Adam Falewicz, Małgorzata Szcześniak

**Affiliations:** 1 Institute of Psychology, University of Opole, 45-040 Opole, Poland; 2 Department of Radiotherapy, Maria Sklodowska-Curie National Research Institute of Oncology, Gliwice Branch, 44-101 Gliwice, Poland; etelka@io.gliwice.pl; 3 Institute of Psychology, University of Szczecin, 70-111 Szczecin, Poland; adam.falewicz@usz.edu.pl (A.F.); malgorzata.szczesniak@usz.edu.pl (M.S.)

**Keywords:** total pain, fear of recurrence, psychological flexibility, mentalization, post-treatment cancer patients

## Abstract

**Background**: The research indicates that painful experiences can significantly affect the fear of cancer recurrence among cancer survivors, which is a distressing concern that influences both physiological and psychological recovery. This cross-sectional study aims to advance our comprehension of the associations between total pain and the fear of recurrence in post-treatment cancer patients by examining two potential mediators: psychological flexibility and mentalization. **Methods:** Three hundred and thirty-five participants (aged 22 to 88, 49.1% female) who had finished their cancer treatment completed self-report assessments of total pain, their fear of recurrence, psychological flexibility, and mentalization. **Results:** The serial mediation analysis showed that all dimensions of total pain were positively and indirectly related to the fear of recurrence through psychological flexibility and mentalization in serial. Additionally, gender was found to moderate these serial mediational effects. **Conclusions:** In line with the psychological flexibility model, personal capacities to face difficult internal/external problems and interpret one’s behavior in motivational terms can counterbalance a patient’s negative emotions and feelings related to the illness. Gender factors also determine the way in which post-treatment cancer patients manage potential future anxiety and fears.

## 1. Introduction

Cancer is affecting an increasing number of people worldwide [1], with approximately 35% of patients experiencing significant psychological distress [2]. Previous studies have shown that a significant proportion of patients who have completed cancer treatment report that managing the fear of cancer recurrence (FCR) is one of their key psychological problems [3,4]. Due to the chronic nature of cancer, pain is a frequent problem in cancer survivors, which strongly affects not only their physical health, but also their overall psychological functioning [5]. Therefore, it is particularly important to examine how factors such as pain and personal resources (e.g., psychological flexibility and mentalization) may increase vs. decrease FCR among those with a history of cancer.

### 1.1. Associations between Total Pain and the Fear of Recurrence

Previous research suggests that cancer pain is a serious health problem among both cancer patients and survivors [6,7]. It affects not only the physical dimensions of the individual, but also psychological, social, and existential ones. The multidimensional construct of total pain was introduced by Saunders as the suffering that encompasses physical, psychological, social, and spiritual sensations interacting upon one another in essential domains of human functioning [8,9].

Research has shown that the dimensions of total pain are negatively related to meaning in life, coping, and psychological well-being in abdominal and pelvic cancer patients undergoing treatment [10]; psychosocial functioning and quality of life in palliative cancer patients; and [11] self-efficacy and illness acceptance in pelvic cancer patients receiving radiotherapy treatment [12]. In addition, associations between the particular dimensions of total pain and psychological outcomes have been found. For example, stronger physiological and psychological pain symptoms are associated with lower levels of physical, social, and emotional well-being among patients treated for cancer pain [13]; greater emotional and social pain are related to lower quality of life and increasing disease severity in hematological malignancies [14]; and more intense spiritual pain is related to poorer coping abilities and higher levels of depression, anxiety, and worry [15]. On the other hand, total pain is positively associated with stress and personal activities aimed at searching for meaning [10,12].

Given that total pain is closely related to the cognitive and emotional processes responsible for patient well-being, it is therefore plausible to expect associations between total pain and FCR. Among cancer survivors, experiencing new or ongoing symptoms of pain has been associated with greater FCR [16]. Pain intensity and pain catastrophizing have also been found to be explicitly responsible for heightened FCR in childhood cancer survivors even if they had not had a relapse for many years [17]. Psychological distress, which is closely related to psychological pain, was found to relate positively to FCR in patients who had experienced different types of cancer [18,19]. Although there have not been any studies to date on the relationship between total pain and FCR, given the psychosocial determinants of FCR [20], such relations seem very plausible. In addition, potential intervention (i.e., mediating) mechanisms should also be examined in the group of post-treatment cancer patients.

### 1.2. Psychological Flexibility and Mentalization as Intervening Variables

There is empirical evidence suggesting that the relationship between total pain and the fear of recurrence may be mediated by factors that enhance patients’ psychological resilience and could facilitate a reinterpretation of the disease. Psychological flexibility and mentalization, whose functional utility in cancer patients has been validated [21,22], offer potentially promising results. Psychological flexibility, which reflects one’s ability to remain in contact with thoughts and emotions during daily activities, was found to mediate the relationships between pain-related disability and life satisfaction among patients suffering from whiplash-associated disorder [23] and health conditions and emotional distress in a group of cancer survivors who were undergoing ACT-based therapy [24]. Psychological flexibility also mediates the relationship between pain intensity and depression in people with chronic pain, which confirms its role as a resilience factor [25]. 

Another factor that can potentially affect the relationship between total pain and the fear of recurrence is mentalization, which represents people’s capacity to perceive and interpret their behavior in terms of intentionally motivated states [26]. This seems likely given the strong tendency of cancer patients to evaluate available motivational resources and their important role in adapting to the disease. Mentalization was found to mediate relationships between stressful life events and emotional dysregulation in a general population [27], as well as between distress and mental health symptoms in patients with psychosomatic problems; a higher level of mentalization was significantly associated with lower depression, anxiety, and somatization [28]. These findings suggest that psychological flexibility and mentalization may underlie the associations between cancer-related pain and the fear of recurrence. However, research has not examined the mediating effects of psychological flexibility and mentalization in post-treatment cancer patients.

The psychological flexibility model, which was proposed within acceptance and commitment therapy (ACT) [29], and proved its usefulness in cancer research [21,24], may advance our conceptualization and provide a theoretical foundation for understanding the complex relations. The model assumes that detrimental internal experiences (e.g., cognitions, feelings, and pain) are an inevitable part of human life and strongly affect psychosocial functioning. However, people have the ability to productively manage these unfavorable experiences and construct healthy and durable behavior changes on the basis of their psychological skills. As a consequence, they can reduce distress and anxiety through using constructive thoughts and behavior. The model includes six core elements, i.e., acceptance, cognitive diffusion, present-focused attention, self as context, values, and committed action, which form a coherent process-oriented structure [30].

Within the psychological flexibility model, painful cognitions and emotions can be positively counteracted and attenuated by the ability to recognize and adapt to various situational demands and to interpret one’s behavior in terms of intentionally motivated states, which, in turn, will lead to less distress and anxiety. In other words, psychological flexibility and functional mentalization can serve as intervening factors by which global perceptions of cancer-related pain, to a lesser extent, affect the potential fear of recurrence. This assumption is supported by empirical studies that have shown that psychological flexibility is a mediator in the association of both anxiety and depression with the fear of recurrence in cancer patients [31], as well as between fatigue and quality of life in people with a cancer diagnosis (either current or previous) [32]. Although, to date, there has not been any research on directly testing mentalization as a mediator in cancer patients, mentalization has been proven to be a frequent mediator in other clinical groups; it mediates the relationship between psychological symptom severity and disabilities in activities and participation in psychotherapy patients who have experienced severe traumatic problems [33], and there is an association between traumatic experiences and depressive symptoms in people suffering from various psychiatric disorders [34].

In addition, gender has been shown to influence the impact of pain on psychological functioning and emotional processes in cancer patients, which may partly be a consequence of women reporting higher FCR [35] and psychological distress [36] than men. Through examining people with different types of cancer, Zajac and al. [37] found that gender moderated the relationship between risk perceptions and cancer worry; absolute risk scores in women were found to be a stronger predictor of worry than in men. Gender is also a moderator of the association between cancer screening and a perceived risk of colon cancer, with the association being stronger among women than among men [38]. In addition, gender is a moderator on disease stage and anxiety and depressive symptoms, with the female gender being associated with higher levels of anxiety and depressive symptoms in advanced disease [39]. However, it should be acknowledged that the number of these studies is relatively small, and that some of these studies did not confirm the moderating role of gender in the relationship of pain with anxiety or worry [40], which may be due to the lack of differences in cancer-related pain between men and women [41]. 

The above findings suggest that the moderating role of gender in the relationship between pain and FCR is dependent on cognitive and emotional factors. Given that both psychological flexibility and mentalization are increasingly recognized as playing a significant role in emotional well-being among cancer patients [42,43], it is highly plausible that they may also attenuate the negative symptoms of total pain and the fear of recurrence. 

**Figure 1:**
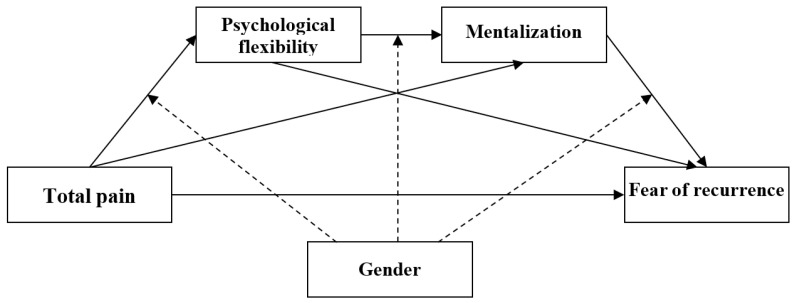
A conceptual model of the serial mediation of psychological flexibility and mentalization in the associations between total pain and the fear of recurrence and moderated mediation by gender.

The current study aims to broaden our understanding of the relation between total pain and FCR by testing potential intervening variables (i.e., psychological flexibility, mentalization, and gender) (Figure 1). Based on previous findings and the psychological inflexibility model, we hypothesized that the dimensions of total pain would be negatively related to psychological flexibility and mentalization, as well as positively related to the fear of recurrence. We also hypothesized that psychological flexibility and mentalization serially mediates the relationship between total pain and the fear of relapse; moreover, it was thought that this relationship would be moderated by gender.

## 2. Methods

### 2.1. Participants and Procedure

The participants comprised 335 adult outpatients diagnosed with cancer between the ages of 22 and 88 (M = 59.78, SD = 13.28) in the southern parts of Poland. The sample included 164 female (49.0%) and 171 male (51.0%) patients. All patients were in the post-treatment stage of cancer. Participants reported numerous types of cancer, with oral cavity and pharynx (N = 75, 22.4%), digestive system (N = 71, 21.2%), and genital and urinary system (N = 52, 15.5%) cancer being the most frequently endorsed. Most participants reported receiving radiotherapy (N = 151, 45.1%), followed by combined therapy (N = 11, 33.1%) and chemotherapy (N = 73, 21.8%). The majority of the sample identified were white or Caucasian (95.6%) and married (81.7%). 

Participants were recruited at cancer treatment clinics during visits with their physician. The following inclusion criteria were set: (1) confirmed stages of cancer from I to III; (2) completion of cancer treatment; (3) lack of serious medical conditions that could significantly distort responses; and (4) sufficient cognitive abilities to complete the questionnaire. Eligible participants were provided with an informed consent form before being invited to complete the questionnaire. A total of 386 patients were approached by research assistants, of whom 29 were not eligible due to the inclusion criteria, 12 refused to participate, and 9 patients returned incomplete tests. A final sample of 335 (86.7%) participants was included in the study. All procedures were conducted in accordance with the approved Institutional Ethics Committee protocol at the first author’s university.

### 2.2. Measures

#### 2.2.1. Total Pain

The Total Pain Questionnaire (TPQ) [44] is a 20-item, self-report assessment of pain and consists of the following four subscales: physical pain (5 items, “As a result of my treatment so far, I am experiencing physical pain”); psychological pain (5 items, “I experience a sense of helplessness and anxiety due to illness”); social pain (5 items, “Being dependent on other people causes me pain”); and spiritual pain (5 items, “I feel an inner, spiritual suffering”). Participants rate each item on a 10-point scale ranging from 1 (not at all) to 10 (very strong), with higher scores indicating greater pain. The TPQ has demonstrated adequate concurrent validity [10]. In the current study, the Cronbach’s alphas for physical pain, psychological pain, social pain, and spiritual pain were 0.85, 0.84, 0.87, and 0.85, respectively.

#### 2.2.2. Psychological Flexibility

The Acceptance and Action Questionnaire [45] is a seven-item measure of psychological flexibility that represents one’s capacity to function in accordance with core goals and values, even while facing difficult internal and external problems (e.g., “My painful experiences and memories make it difficult for me to live a life that I would value”). Participants rate each item on a seven-point scale ranging from 1 (never true) to 7 (always true), with higher scores indicating greater psychological flexibility. In the current study, Cronbach’s alpha was 0.90.

#### 2.2.3. Mentalization

The Reflective Functioning Questionnaire (RFQ) is an eight-item scale measuring mentalization, which is understood as the capacity to perceive and interpret a person’s own and others’ behavior in terms of intentionally motivated states (e.g., “People’s thoughts are a mystery to me”) [46]. The items are rated on a seven-point Likert scale ranging from 1 (completely disagree) to 7 (completely agree). The Polish version of the RFQ [47] was used, which measures mentalization only as a unidimensional construct (the original scale includes two subscales: the degree of uncertainty and the certainty concerning mental states). Higher scores indicate greater mentalization. In the current study, the Cronbach’s alpha was 0.78.

#### 2.2.4. The Fear of Recurrence

The Cancer Worry Scale (CWS) [48] is an eight-item scale that assesses concerns about developing cancer again and the effect of these concerns on daily life (e.g., “How often have you thought about your chances of getting cancer again?”). Participants rate each item on a four-point scale from 1 (never) to 4 (almost always). Higher scores indicate greater concern about cancer recurrence. The Cronbach’s alpha for the current study was 0.87.

### 2.3. Statistical Analysis

Analyses were conducted using SPSS version 28 with the PROCESS macro [49]. A priori power analysis was conducted to assess whether there was a sufficient number of participants; G*Power 3.1 was used in accordance with Faul et al.’s recommendations [50] (a test power of 1 − β = 0.80, α = 0.05). The results indicated that 304 participants would be required to obtain an effect size of 0.04. In adding a 10% non-response rate, the final sample size was 335.

Statistical analyses were conducted in several steps. First, as our study was based on mediation analysis, Harman’s single-factor test was used to check the possibility of common method variance [51]. Its results demonstrated satisfactory indicators, where there were 18 different factors, with the first unrotated factor explaining only 18.48% of the variance, which precluded the common method variance error. Second, the variance inflation factor (VIF) was 1.24, which excluded any potential multicollinearity. Third, descriptive statistics and bivariate correlations were calculated for all of the variables. Finally, mediation analysis (Model 4) with two parallel mediators (psychological flexibility and mentalization) and moderated mediation analysis (gender as a moderator) (Model 92) were applied to examine the direct and indirect effects of total pain on the fear of recurrence (bootstrapping with 10,000 samples; 95% bias-corrected confidence intervals) [49].

## 3. Results

### 3.1. Descriptive and Preliminary Statistics

First, descriptive and bivariate statistics were calculated (Table 1). Age was only negatively correlated with physical and social pain, as well as psychological flexibility. All dimensions of total pain, i.e., physical, psychological, social, and spiritual, were positively associated with the fear of recurrence but negatively associated with psychological flexibility. Physical and psychological pain, but not social and spiritual pain, were negatively associated with mentalization. Both psychological flexibility and mentalization were negatively associated with the fear of recurrence.

### 3.2. Serial Mediation Model

Serial mediation analysis (Model 6) was conducted to examine whether psychological flexibility and mentalization would mediate the relationships between the dimensions of total pain and the fear of recurrence, respectively (Table 2).

**Figure 2:**
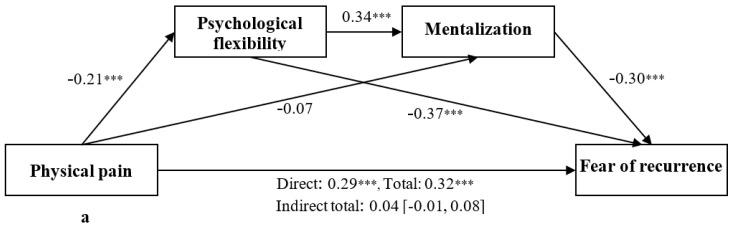

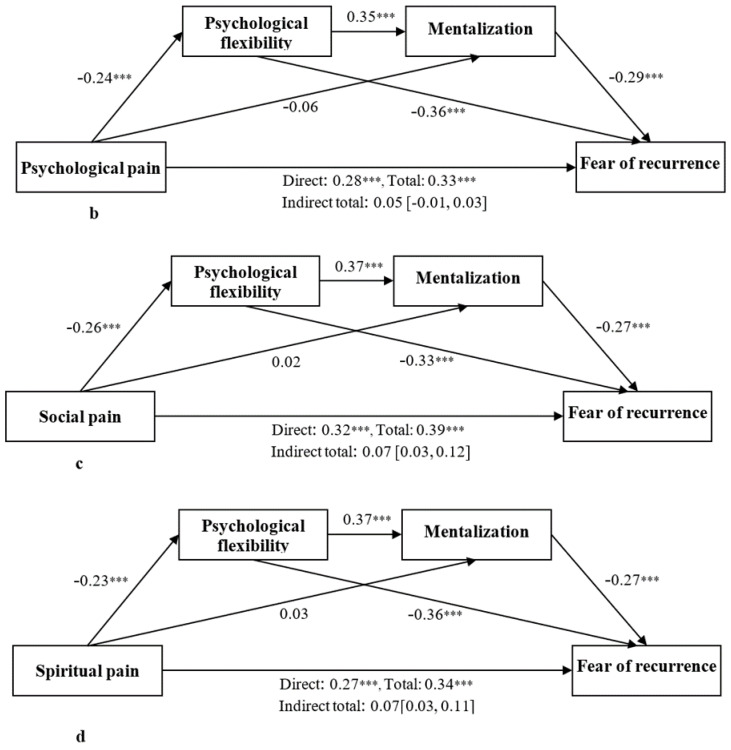
(**a**–**d**) Serial mediation models for psychological flexibility and mentalization in associations between the physical dimension of total pain and the fear of recurrence. *** *p* < 0.001.

The examination of direct effects revealed that physical, psychological, social, and spiritual pain were negatively related to psychological flexibility but were unrelated to mentalization (Figure 2a–d). Psychological flexibility was positively related to mentalization and negatively related to the fear of recurrence, while mentalization was negatively related to the fear of recurrence. In contrast, the four dimensions of total pain, namely physical, psychological, social, and spiritual, were positively related to the fear of recurrence.

As hypothesized, there were significant serial mediational effects of psychological flexibility and mentalization on the relations between the dimensions of total pain and the fear of recurrence (Table 2). Specifically, the examination of direct effects showed that lower levels of physical, psychological, social, and spiritual pain were related to a higher level of psychological flexibility and consequently to higher mentalization, which, in turn, was related to a lower fear of recurrence (Figure 2a–d). In addition, some single mediational effects turned out to be significant. All single mediational effects of psychological flexibility in associations between the dimensions of total pain and the fear of recurrence were significant, but the mediational effects of mentalization were found to be non-significant. This reflected the stronger mediating effect of psychological flexibility compared to mentalization.

In addition, it was also checked whether age could be a confounding factor in our analysis. The results of mediation analysis with the covariant option (i.e., age as a covariant) showed that age did not play a significant role in the mediating relations between the dimensions of total pain and the fear of recurrence (i.e., in all cases, the range of LLCI and ULCI levels included zero). The effect of the treatment types on the mediating relations were also examined, yet they turned out to be non-significant (i.e., in all cases, the range of LLCI and ULCI levels included zero).

The results of the indirect effects contrast showed that, for physical and psychological pain, the indirect effect through psychological flexibility was significantly stronger than the indirect effect through mentalization, and the serial indirect effect was also significant stronger through psychological flexibility and mentalization. Additionally, the serial indirect effect through psychological flexibility and mentalization was stronger than the indirect effect through mentalization in the case of social and spiritual pain.

As our study utilized a cross-sectional design, the alternative models, including the reverse orders of mediators (i.e., total pain → mentalization → psychological flexibility → the fear of recurrence), were tested in serial. However, the results of the indirect effects turned out to be mostly non-significant (e.g., for physical and psychological pain, the indirect effects through mentalization and psychological flexibility on the fear of recurrence were IE = −0.01; CI = −0.04 to 0.03, and IE = −0.007; and CI = −0.03 to 0.07, respectively).

### 3.3. Moderated Mediation Model: Gender Moderation

The current analysis tested whether the relationship between total pain and the fear of recurrence through psychological flexibility and mentalization is moderated by gender. Model 92 (moderation effect on all pathways) was chosen as the most advanced because it allowed us to examine whether all the indirect effects of total pain on the fear of recurrence—in both single (X→M1→Y, X→M2→Y) and serial mediations (X→M1→M2→Y)—are contingent on gender [48]. The statistical significance of the ‘Index of Moderated Mediation’ effect was assessed along with the conditional indirect effects across all four dimensions of total pain.

The results showed some significant indirect effects and moderated mediation indices (Table 3). Gender did not moderate any of the mediational effect of physical pain on the fear of recurrence, although three of the six indirect effects (IE) were found to be significant: the effect of physical pain through psychological flexibility on the fear of recurrence for both females (IE = 0.02; CI = 0.01 to 0.03) and males (IE = 0.04; CI = 0.02 to 0.06), and the effect of physical pain through psychological flexibility and mentalization on the fear of recurrence for males (IE = −0.02; CI = −0.02 to −0.01). For psychological pain as an independent variable, gender was found to significantly moderate its mediational effect on the fear of recurrence through mentalization. Specifically, for men, the conditional IE was non-significant (IE = 0.001; CI = −0.01 to 0.01), whereas, for women, the conditional IE was stronger and statistically significant (IE = −0.03; CI = −0.04 to −0.01). Gender also moderated the serial mediational effect of psychological pain on the fear of recurrence through psychological flexibility and mentalization. The conditional IE was only significant for women (IE = −0.02; CI = −0.02 to −0.01). For social pain, gender was found to moderate the single mediational effect through psychological flexibility, with women having stronger conditional IE (IE = 0.05; CI = 0.03 to 0.07) than men (IE = 0.01; CI = 0.01 to 0.03). Gender was also found to moderate the serial mediational effect of social pain on the fear of recurrence through psychological flexibility and mentalization. Specifically, for men, the conditional IE was non-significant (IE = −0.002; CI = −0.01 to 0.002), whereas, for women, the conditional IE was stronger and statistically significant (IE = −0.04; CI = −0.03 to −0.01). Finally, gender was found to moderate the serial mediational effect of spiritual pain through psychological flexibility and mentalization; the conditional IE for women was stronger and more significant (IE = −0.04; CI = −0.03 to −0.01) than for men (IE = −0.002; CI = −0.01 to 0.01). 

In general, psychological flexibility and mentalization were found to be more significant mediators in the relationships of psychological, social, and spiritual pain with the fear of recurrence in men than in women. However, there was no meaningful difference in the mediating effects of psychological flexibility and mentalization in the relationships between physical pain and the fear of recurrence between men and women. Gender was found to moderate the serial mediational effect of psychological, social, and spiritual pain on the fear of recurrence through psychological flexibility and mentalization. In addition, to check whether there were any differences in the relationships examined in terms of cancer types, we performed moderation analyses in which the moderator was the type of cancer. However, the results showed that the type of cancer did not play a significant role in these relationships (*p* > 0.05).

## 4. Discussion

Cancer-related pain and FCR are significant problems among patients who have completed their treatment. To date, our understanding of the variables and psychological mechanisms underlying these relations has been limited. The aim of this study was to investigate the mediating role of psychological flexibility and mentalization as serial mediators, as well as to better understand the moderating function of gender among post-treatment cancer patients in the relationship between total pain and FCR.

### 4.1. Associations among Total Pain, Psychological Flexibility, Mentalization, and the Fear of Recurrence

In our sample, all the dimensions of total pain, i.e., physical, psychological, social, and spiritual, were positively related to the fear of recurrence. These results are consistent with previous studies in which symptoms of pain (new or ongoing) are related to higher levels of FCR in cancer survivors [16], and where pain intensity and pain catastrophizing are predictors of greater FCR in childhood cancer survivors [17]. Our study expands upon previous findings by showing that, in post-treatment cancer patients, not only do physical or psychological aspects of pain influence the fear of potential recurrence, but the social or spiritual experiences of pain also activate such negative feelings of anxiety. In people with cancer, suffering thus encompasses all of the person’s physical, psychological, social, and spiritual struggles, thereby generating thoughts and emotions related to the potential development of this disease.

In addition, all of the dimensions of total pain were found to be negatively related to psychological flexibility, and physical and psychological pain were found to be negatively related to mentalization. Our results are in line with those in previous research, where it has been demonstrated that cancer patients with higher levels of psychological flexibility experience less emotional distress [24]. In addition, those patients with psychosomatic problems and higher mentalization were found to have lower depression, anxiety, and somatization [28]. These relationships seem particularly interesting as they indicate that various dimensions of cancer-related pain tend to undermine patients’ capacity to face challenging internal/external problems and to efficiently understand their own mental states, as well as those of others. This interpretation is supported by research showing that painful physical and psychological feelings associated with cancer affect the way patients think about their ability in maintaining internal integration and coping with adversity [24,52]. In patients with advanced, chronic illness, pain is thus a multidimensional symptom that affects their cognitions and feelings.

### 4.2. The Mediating and Moderating Effects of Psychological Flexibility, Mentalization, and Gender

The key findings of the current study showed the serial mediational effects of psychological flexibility and mentalization on the relationships between the dimensions of total pain and the fear of recurrence. Specifically, patients who experience more pain in physical, psychological, social, and spiritual domains as a result of their cancer or cancer treatment are characterized by a higher ability in managing problematic internal/external situations, as well as in interpreting their behavior in terms of motivational states, which, in turn, is associated with less concern about developing cancer again. The serial nature of this mediation means that recognizing and adapting to various situational demands precedes interpreting one’s own actions. Additionally, the contrast in the indirect effects showed that individuals who experience more cancer-related total pain tend to rely more on their capacity to handle adverse problems on the basis of core goals and values (psychological flexibility) than to cognitively understand their own and others’ mental states (mentalization). Analyzing them both together in the context of the FCR is therefore justified.

These findings highlight the role of personal abilities based on motivational and cognitive states as potential mechanisms underlying the association between cancer-related total pain and FCR. They are consistent with previous studies that have shown the conceptual utility of psychological flexibility and mentalization in cancer patients [21,22]. The findings were also found to be in accordance with the mediating role of psychological flexibility in the association between pain intensity and depression in non-cancer patients [25], as well as of mentalization in the association between distress and mental health symptoms in patients with psychosomatic problems [28]. They also offer support for the notion that associations between total pain and FCR have an indirect rather than a direct character, especially for individuals who have completed their cancer treatment.

At the same time, taken together, these results expand our understanding of the complex relationship between total pain and FCR as they reveal two significant aspects of emotional anxiety and adversity associated with cancer. First, for post-treatment cancer patients, different dimensions of total pain and the fear of relapse do not occur as separate entities in an “existential vacuum” but are rather functionally interconnected on the basis of the ability to manage challenging or adverse situations and to comprehend one’s own mental states. This notion is supported by earlier research that showed the mediating effects of psychological flexibility and mentalization between pain outcomes and detrimental emotional and health symptoms in cancer [24] and non-cancer [27] patients. In other words, what determines the final character of the relationship between total pain and FCR are flexible and mentalizing approaches. 

Second, the serial character of the mediation (i.e., the psychological flexibility–mentalization mediation) suggests that, despite the cross-sectional nature of our study, identifying and adapting to challenging illness conditions precedes the modes of interpreting a patient’s own behavior. Consequently, patients who experience a high level of total pain will first try to maintain a balance between their important life domains and then to construe their own actions in terms of intentional motivation, which, in turn, will lead to a reduced fear of relapse. This interpretation received some confirmation within the psychological flexibility model, which assumes that individuals can alleviate painful cognitions and emotions (first, by recognizing and harmonizing the various situational demands of life, and then, by developing patterns of effective action related to chosen values) [21,30]. Psychological flexibility and mentalization are thus mediating mechanisms through which patients can balance their negative emotions and feelings about the disease.

By expanding upon previous studies on the moderating role of gender in the relationship between pain and emotional processes in cancer patients [37,38], this study specified that gender moderates the serial mediational effect of the three dimensions of total pain (i.e., psychological, social, and spiritual) on the fear of recurrence through psychological flexibility and mentalization. In all cases, the effect was stronger for women than men. These results confirm prior research on cancer patients in which gender was a moderator in the relationship between risk perceptions and cancer worry, with women obtaining stronger effects than men [39], as well as in the association between disease stage and anxiety, with women reporting higher levels of anxiety [41]. This may be due to a greater tendency in women to react emotionally and worry about the disease and its potential consequences [37], as well as experiencing a statistically higher level of FCR than men [38]. Another additional explanation for this may relate to women’s greater ability to be aware of unfavorable thoughts and emotions about cancer, as well as their greater tendency to self-analyze.

Despite this study’s contribution to the current research on pain and FCR, there are some limitations that need to be addressed. First, our study applied a cross-sectional design that precludes any temporal relationships, yet the alternative models with a reversed sequence of mediators, in serial, were found to be not significant. Future research should consider examining longitudinal relations among the variables tested in our study in order to definitively confirm their direction. Second, the current study measured mentalization conceptualized as a unidimensional construct [37]. Given the existence of other multidimensional measures of mentalization [53], their future use could expand our knowledge about the mediating role of mentalization. Finally, our study used a single tool to measure FCR, so prospective studies should use multidimensional measures to estimate the various fear and anxiety factors associated with cancer recurrence. 

## 5. Conclusions

Notwithstanding these limitations, the present study broadens our understanding of mediating factors and psychological mechanisms in the relation between cancer-related total pain and the fear of cancer recurrence. Importantly, it established the significant role of psychological flexibility and mentalization in the association between total pain and FCR in post-treatment cancer patients. Our study also has important clinical implications for the work of physicians, psychologists, or nurses. First, it highlights the important role of a specialized approach based on personal resources in treating the various dimensions of pain and illness anxiety. Second, it emphasizes the significance of using motivational factors (e.g., one’s abilities or coping skills), which can reduce the fear of recurrence or lower the negative emotions associated with illness. These approaches can improve the treatment and psychosocial functioning of cancer patients who have undergone treatment.

## Figures and Tables

**Table 1:** Correlations among age, total pain, the fear of recurrence, psychological flexibility, and mentalization.

Variables	M	SD	1.	2.	3.	4.	5.	6.	7.
1. Age	59.78	13.28	-						
2. Physical pain	3.03	2.18	−0.20 ***	-					
3. Psychological pain	3.31	2.16	−0.10	0.50 ***	-				
4. Social pain	2.61	1.95	−0.12 *	0.57 ***	0.70 ***	-			
5. Spiritual pain	2.15	1.99	−0.10	0.47 ***	0.58 ***	0.76 ***	-		
6. The fear of recurrence	1.98	0.70	−0.09	0.32 ***	0.33 ***	0.39 ***	0.34 ***	-	
7. Psychological flexibility	4.92	1.06	−0.11 *	−0.21 ***	−0.24 ***	−0.26 ***	−0.23 ***	−0.32 ***	-
8. Mentalization	4.62	0.91	−0.09	−0.14 **	−0.14 **	−0.07	−0.05	−0.13 *	0.36 ***

* *p* < 0.05; ** *p* < 0.01; and *** *p* < 0.001.

**Table 2:** Standardized indirect effects of the dimensions of total pain on the fear of recurrence through psychological flexibility and mentalization, as well as on the specific indirect effect contrasts for the paths.

Variables				
Indirect effects for physical pain	Effect	SE	LLCI	ULCI
PhP → PF → FoR (Ind 1)	0.08	0.02	0.04	0.12
PhP → Me → FoR (Ind 2)	−0.02	0.02	−0.05	0.01
PhP → PF → Me → FoR (Ind 3)	−0.02	0.01	−0.04	−0.01
Total	0.04	0.02	−0.01	0.08
Indirect effects contrast				
C 1 = Ind 1–Ind 2	0.10	0.03	0.05	0.16
C 2 = Ind 1–Ind 3	0.10	0.03	0.05	0.15
C 3 = Ind 2–Ind 3	0.01	0.02	−0.04	0.04
Indirect effects for psychological pain				
PsP → PF → FoR (Ind 1)	0.09	0.02	0.05	0.13
PsP → Me → FoR (Ind 2)	−0.02	0.02	−0.05	0.01
PsP → PF → Me → FoR (Ind 3)	−0.03	0.01	−0.04	−0.01
Total	0.05	0.02	−0.01	0.09
Indirect effects contrast				
C 1 = Ind 1–Ind 2	0.10	0.03	0.05	0.17
C 2 = Ind 1–Ind 3	0.11	0.03	0.05	0.17
C 3 = Ind 2–Ind 3	0.01	0.02	−0.03	0.05
Indirect effects for social pain				
SoP → PF → FoR (Ind 1)	0.03	0.02	0.05	0.14
SoP → Me → FoR (Ind 2)	0.01	0.01	−0.02	0.04
SoP → PF → Me → FoR (Ind 3)	−0.03	0.01	−0.04	−0.01
Total	0.07	0.02	0.03	0.12
Indirect effects contrast				
C 1 = Ind 1–Ind 2	0.08	0.03	0.03	0.14
C 2 = Ind 1–Ind 3	0.11	0.03	0.06	0.17
C 3 = Ind 2–Ind 3	0.04	0.02	0.01	0.07
Indirect effects for spiritual pain				
SpP → PF → FoR (Ind 1)	0.08	0.02	0.04	0.13
SpP → Me → FoR (Ind 2)	0.01	0.01	−0.02	0.04
SpP → PF → Me → FoR (Ind 3)	−0.02	0.01	−0.04	−0.01
Total	0.07	0.02	0.03	0.11
Indirect effects contrast				
C 1 = Ind 1–Ind 2	0.07	0.03	0.02	0.13
C 2 = Ind 1–Ind 3	0.10	0.03	0.05	0.16
C 3 = Ind 2–Ind 3	0.03	0.02	0.01	0.07

PhP—physical pain; PsP—psychological pain; SoP—social pain; SpP—spiritual pain; PF—psychological flexibility; Me—mentalization; and FoR—the fear of recurrence.

**Table 3:** Moderated mediation estimates for the fear of recurrence outcomes.

*Moderated Mediation Index*	Effect (SE)	LLCI	ULCI
PhP → PF → FoR	0.02 (0.01)	−0.01	0.04
PhP → Me → FoR	−0.01 (0.01)	−0.02	0.01
PhP → PF → Me → FoR	−0.01 (0.01)	−0.02	0.001
PsP → PF → FoR	0.02 (0.01)	−0.01	0.04
PsP → Me → FoR	−0.04 (0.01)	−0.05	−0.01
PsP → PF → Me → FoR	−0.05 (0.01)	−0.02	−0.01
SoP → PF → FoR	0.05 (0.02)	0.01	0.05
SoP → Me → FoR	−0.01 (0.01)	−0.02	0.02
SoP → PF → Me → FoR	−0.06 (0.01)	−0.03	−0.01
SpP → PF → FoR	0.02 (0.01)	−0.01	0.05
SpP → Me → FoR	0.01 (0.01)	−0.01	0.03
SpP → PF → Me → FoR	−0.05 (0.01)	−0.02	−0.01

PhP— physical pain; PsP— psychological pain; SoP— social pain; SpP— spiritual pain; PF— psychological flexibility; Me— mentalization; and FoR—the fear of recurrence.

## Data Availability

The data presented in this study are available at OSF Home: https://osf.io/5j2hd/.
